# Ceftazidime-avibactam and intrapleural amikacin therapy for extensively drug-resistant *Pseudomonas aeruginosa* thoracic empyema: A case report

**DOI:** 10.1097/MD.0000000000029467

**Published:** 2022-06-17

**Authors:** Tzu-Ting Chen, Shu-Mei Chen, Hsin-Yi Liu

**Affiliations:** a Department of Pharmacy, Taipei Medical University Hospital, Taipei Medical University, Taipei, Taiwan; b Department of Neurosurgery, Taipei Medical University Hospital, Taipei Medical University, Taipei, Taiwan; c Department of Surgery, School of Medicine, Taipei Medical University, Taipei, Taiwan; d Division of Infection Diseases, Department of Internal Medicine, Taipei Medical University Hospital, Taipei, Taiwan.

**Keywords:** case report, ceftazidime-avibactam, *Pseudomonas aeruginosa*, thoracic empyema

## Abstract

**Introduction:**

Thoracic empyema and concomitant bronchopleural fistula are serious complications of pneumonia. The treatment of empyema caused by extensively drug-resistant *Pseudomonas aeruginosa* (XDR-PA) has become increasingly challenging.

**Patient's concerns and important clinical findings:**

A 57-year-old woman with controlled schizophrenia developed hospital-associated bacterial pneumonia secondary to *P. aeruginosa* on day 13 of hospitalization for brain meningioma surgery.

**Diagnosis:**

Chest radiography and computed tomography revealed right-sided necrotizing pneumonia with pneumothorax, a focal soft tissue defect over the right lower chest wall, and a mild right-sided encapsulated pleural effusion with consolidation. XDR-PA was isolated on empyema cultures.

**Interventions:**

The patient was treated with intrapleural amikacin as a bridge to video-assisted thoracoscopic surgery, followed by novel ceftazidime-avibactam therapy.

**Outcomes:**

On the 104th day of admission, the patient underwent chest wall debridement and closure. The patient was discharged on day 111. Twenty-eight days after discharge, there were no observable sequelae of empyema.

**Conclusion:**

Although the minimum inhibitory concentration of ceftazidime-avibactam for XDR-PA is relatively high (8 mg/L), this report emphasizes the efficacy of ceftazidime-avibactam treatment for XDR-PA empyema, as well as the importance of source control.

## 1 Introduction

*Pseudomonas aeruginosa* (PA) is a common pathogen in hospital/ventilator-associated bacterial pneumonia (HABP/VABP). The worldwide prevalence of extensively drug-resistant PA (XDR-PA) with susceptibility to aminoglycosides and polymyxins is well known. Thoracic empyema and bronchopleural fistulas are serious complications of pneumonia. Ceftazidime-avibactam has been approved for use in adults with HABP/VABP and other aerobic gram-negative infections. It is potent against multidrug-resistant PA (MDR-PA).^[1]^ Large-scale surveillance studies suggest that ceftazidime-avibactam should be used to treat hospitalized patients with carbapenem-resistant *Enterobacterales* and MDR-PA infections who have limited treatment options.^[2]^ Taiwanese health insurance policies have approved ceftazidime-avibactam for the treatment of HABP; 84–97% of clinical isolates had a ceftazidime/avibactam minimum inhibitory concentrations (MIC) of ≤ 8 μg/mL. The penetration of ceftazidime/avibactam in empyema or fistula had not been well established; therefore, it was unavailable in our formulary at the time the current patient was being treated.

## 2 Case report

**Figure 1:**
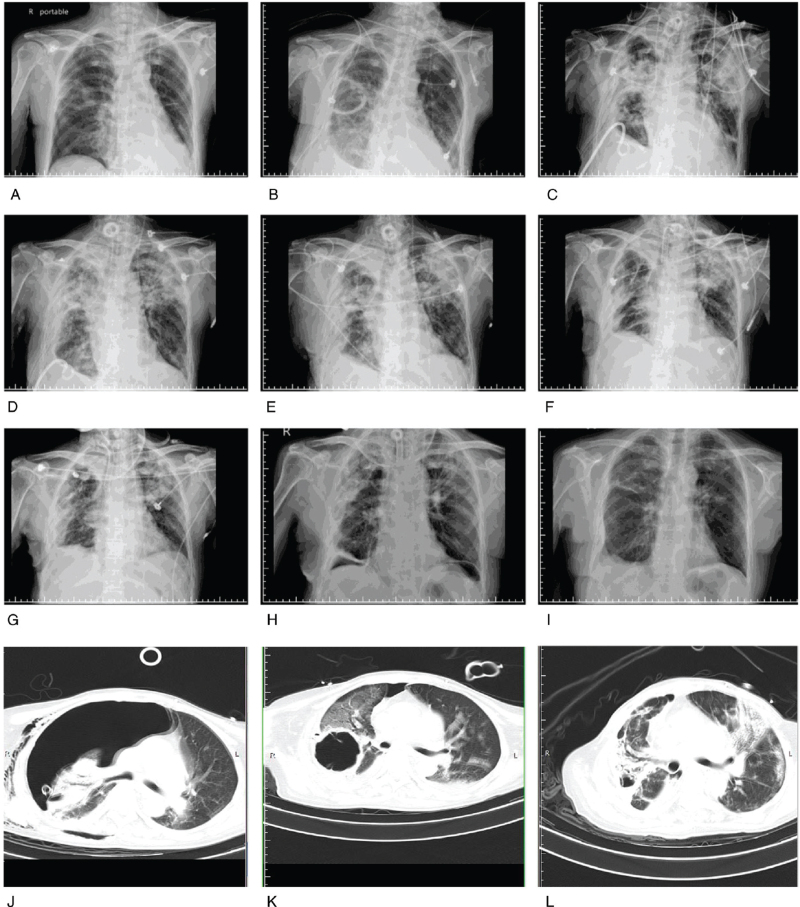
The patient's serial chest radiographs. Day of hospitalization: (A) D 24, (B) D 29, (C) D 36, (D) D 45, (E) D 49, (F) D 73, (G) D 80, (H) D 104, and (I) 28 days after discharge. Chest computed tomography scan on D 25 (J), D 31 (K), and D 57 (L).

A 57-year-old woman with controlled schizophrenia suddenly developed dizziness with muscle weakness at home. She was admitted for brain meningioma surgery after being diagnosed with a large hypothalamus-compressing tumor in the emergency room. HABP secondary to PA developed on hospital day (D) 13. Empirical intravenous therapy was changed from amoxicillin-clavulanate to extended piperacillin-tazobactam infusion and inhaled colistin. Over the ensuing days, her antibiotic regimen was changed multiple times due to persistent breakthrough fever. First, piperacillin-tazobactam was replaced with parenteral colistin and extended infused with meropenem, and inhaled colistin was continued (Fig. 1A). The minimum inhibitory concentrations (MICs) and sensitivities (S) of piperacillin-tazobactam, ciprofloxacin, meropenem, and ceftazidime were ≤4/4 (S), <0.5 (S), 0.25 (S), and 2 (S) mg/L, respectively (Table 1). Chest computed tomography (CT) on D 25 (Fig. 1J) revealed right necrotizing pneumonia with pneumothorax. The patient remained febrile over the following days, accompanied by an increase in oxygen demand. Therefore, parenteral colistin was replaced with high-dose ciprofloxacin to improve penetration. Repeat chest radiography and CT on D 31 demonstrated empyema (Fig. 1B, 1K). Carbapenem-resistant pathogens were suspected. Therefore, meropenem was replaced with high-dose piperacillin-tazobactam with extended infusion, and ciprofloxacin was continued. The pigtail catheter then began having purulent drainage, and the patient developed an alveolar pleural fistula with severe sepsis, 10 days later (Fig. 1C). Carbapenem-resistant PA was isolated from empyema cultures. Within five days of initiating meropenem therapy, the MICs of piperacillin-tazobactam, meropenem, and ceftazidime increased and persisted at 16/4 (S), 4 [resistant], and 8 (S) mg/L, respectively. However, the MICs of ciprofloxacin and amikacin remained at <0.5 (S) and <8 (S) mg/L, respectively (Table 1). The patient's instability due to severe sepsis prevented surgical decortication. Salvage combination therapy was initiated with high-dose extended meropenem infusion, high-dose ciprofloxacin, and adjunctive intrapleural amikacin (500 mg with urokinase twice daily). This therapy resolved sepsis and permitted video-assisted thoracoscopic surgery decortication. (Fig. 1D). Video-assisted thoracoscopic surgery and Eloesser flaps were performed on D 49 (Fig. 1E).

**Table 1:** Culture and antimicrobial susceptibility testing results.

			Antimicrobial susceptibility testing results^∗^										
Sample type	Hospital day	Culture result	Gentamicin	Amikacin	Ceftazidime	Imipenem	Cefepime	Ciprofloxacin	Levofloxacin	Colistin	Meropenem	Piperacillin–tazobactam	Ceftazidime–avibactam
Sputum	22	*Pseudomonas aeruginosa*	S (≤2)	S (≤8)	S (2)	S (2)	S (2)	S (≤0.5)	S (≤1)	S (≤1)	S (≤0.25)	S (≤4/4)	-
Pleural Fluid	31	*Pseudomonas aeruginosa*	S (≤2)	S (≤8)	S (8)	R (>4)	S (8)	S (≤0.5)	I (4)	S (≤1)	R (>4)	S (16/4)	-
Empyema	51	CR-*Pseudomonas aeruginosa*	I (8)	S (16)	R (>16)	R (>4)	R (>16)	R (>2)	R (>4)	S (≤1)	R (>4)	R (>64/4)	-
Sputum	69	CR-*Pseudomonas aeruginosa*	S (≤2)	S (≤8)	R (>16)	R (>4)	I (16)	R (>2)	R (>4)	S (≤1)	R (>4)	I (64/4)	8 (S)

CR = carbapenem-resistant.

One week postoperatively, the patient developed fever with pus-like pleural drainage (D 51). Empyema cultures isolated XDR-PA that was susceptible only to amikacin and colistin with MICs of 16 (S) and ≤1 (S) mg/L, respectively. Ciprofloxacin was continued for 25 days (MIC ranged from <0.5 to 2 mg/L) (Table 1), and high-dose meropenem was replaced with colistin combined with high-dose fosfomycin due to meropenem-related leukopenia. Repeat chest CT revealed a focal soft tissue defect over the right lower chest wall and a mild right encapsulated pleural effusion with consolidation (Fig. 1L). Fosfomycin was discontinued because of eruptions all over her body.

**Figure 2:**
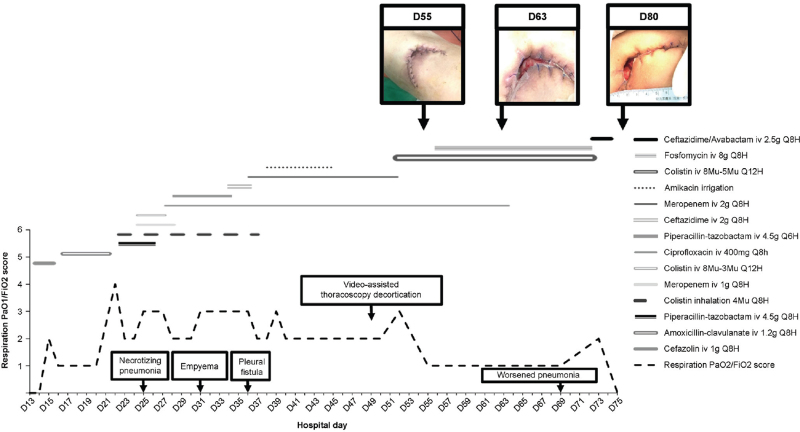
The total treatment course and photographs of the wound.

We proceeded with the emergency procurement of ceftazidime-avibactam. The bioMerieux E-test revealed an MIC of 8 mg/L. A second treatment course of intrapleural irrigation was commenced for fever and purulent drainage while we attempted to procure ceftazidime-avibactam. Ceftazidime-avibactam monotherapy was initiated on D 63. Ciprofloxacin was stopped after the patient remained afebrile for six consecutive days. On D 69, the patient had a new onset of fever and worsening pneumonia due to an XDR-PA infection. (Fig. 1F). On the sixth day after the initiation of ceftazidime-avibactam therapy, serial imaging demonstrated obvious improvement from D 69 until the day of sepsis resolution. Her progress was evidenced by an improved procalcitonin level (0.071 ng/mL) (Fig. 1G) and decreased purulent drainage. Sputum cultures on D 85 demonstrated eradication of PA. The patient underwent chest wall debridement and closure on D 104 (Fig. 1H) and was discharged on D 111. Chest radiography was performed 28 days after discharge (Fig. 1I). The complete treatment course and photographs of the wounds are shown in Fig. 2.

## 3 Discussion

In our case, the patient remained critically septic with persistent fever, despite serial combination therapy with antipseudomonal agents. Resistance to antimicrobial agents progressively increased in serial culture results due to failed source control with empyema and bronchopleural fistula development. Surveillance studies have suggested that ceftazidime-avibactam and ceftolozane-tazobactam retain activity against >85% of MDR-PA isolates.^[3]^ Ceftazidime-avibactam had received approval in Taiwan only three months prior, so it was not readily available to us and required an emergency application to procure.

We are reporting the first use of ceftazidime-avibactam in our hospital. Although the E-test results showed a relatively high MIC (8 mg/L), combination therapy with ceftazidime-avibactam and colistin showed no evidence of better clinical outcomes or suppression of resistance.^[4,5]^ Therefore, we chose ceftazidime-avibactam as a monotherapy.

We performed amikacin pleural irrigation before ceftazidime-avibactam became available. Small comparative studies have reported favorable results when intrapleural antimicrobial therapy is added to adequate pleural drainage and systemic antimicrobial therapy.^[6,7]^ In our case, intrapleural irrigation successfully stabilized the patient's condition as a bridge to the surgery.

To the best of our knowledge, this is the first report of XDR-PA thoracic empyema successfully treated after 21 days with ceftazidime-avibactam. The outcome of this case highlights the importance of source control and eradication, and the efficacy of ceftazidime-avibactam for treating XDR-PA empyema.

## 4 Informed consent

The investigators adhered to policies to protect human subjects. The patient provided written informed consent for the publication of clinical and imaging details. The study was approved by the Joint Institutional Review Board of Taipei Medical University (TMU-JIRB No.: N202102019).

## Acknowledgments

This was a successful multispecialty collaboration. We are grateful to the pulmonary and critical care physician (Kevin Shu-Leung Lai), thoracic surgeon (Cheng-Chin Chung), surgical intensive care unit nurse practitioners, and registered nurses. The authors would like to disclose that they had no writing assistance.

## Author contributions

Tzu-Ting Chen was responsible for investigation and resources. Hsin-Yi Liu and Tzu-Ting Chen were responsible for conceptualizing the study. Shu-Mei Chen and Hsin-Yi Liu were responsible for organizing and coordinating resources. All authors contributed to the writing of the final manuscript.

**Conceptualization:** Hsin-Yi Liu, Tzu-Ting Chen.

**Investigation:** Tzu-Ting Chen.

**Resources:** Hsin-Yi Liu, Shu-Mei Chen, Tzu-Ting Chen.

**Writing – original draft:** Hsin-Yi Liu, Shu-Mei Chen, Tzu-Ting Chen.

**Writing – review & editing:** Hsin-Yi Liu, Shu-Mei Chen.
